# NAFLD, Insulin Resistance, and Diabetes Mellitus Type 2

**DOI:** 10.1155/2021/6613827

**Published:** 2021-02-17

**Authors:** Marinko Marušić, Matej Paić, Mia Knobloch, Ana-Marija Liberati Pršo

**Affiliations:** ^1^Department of Haepatogastroenterology, University Hospital Sveti Duh, Zagreb, Croatia; ^2^Faculty of Medicine, J.J. Strossmayer University of Osijek, Osijek, Croatia; ^3^Faculty of Health Studies, University of Rijeka, Rijeka, Croatia; ^4^Department of Endocrinology and Diabetology, University Hospital Sveti Duh, Zagreb, Croatia

## Abstract

Nonalcoholic fatty liver disease is a condition defined by fat accumulation in hepatocytes not promoted by excessive alcohol consumption. It is highly prevalent and is strongly associated with insulin resistance, metabolic syndrome, and diabetes type II. Insulin resistance plays a crucial role in the multifactorial etiopathogenesis of this condition leading to accumulation of free fatty acids in the liver cells, thus causing lipotoxicity, inflammation, and fibrosis. In this review, we will focus on currently known pathogenesis of nonalcoholic fatty liver disease. Numerous investigation strategies are available to establish the diagnosis, from biochemical markers and ultrasound to various molecular and advanced imaging techniques and liver biopsy. Prevention is crucial. However, effective and promising therapies are strongly demanded.

## 1. Introduction

Nonalcoholic fatty liver disease (NAFLD) is defined as fat accumulation in the liver cells in patients without excessive alcohol consumption. To confirm the diagnosis, more than 5% of hepatocytes must contain lipid droplets when analyzed on light microscopy [1]. NAFLD can be further divided into nonalcoholic fatty liver (NAFL) and nonalcoholic steatohepatitis (NASH) depending on whether or not inflammation is present. Although it was historically, and perhaps even today in some cases, considered as benign, this condition must be taken seriously as it can lead to liver fibrosis, cirrhosis, hepatocellular carcinoma (HCC), and liver failure. What is worrying is that we see significant rise in prevalence not only in NAFLD but as well in other conditions accompanying this disease [2]. This has led NAFLD to become the most prevalent liver disorder in the last few decades [3]. According to recent statistics, there will be even greater rise in liver cirrhosis and other sequels of liver steatosis and steatohepatitis. At the moment, NAFLD is the second cause of liver disease in patients requiring liver transplantation in the USA and is expected to become number one cause for liver transplantation [4]. There is plethora of evidence that NAFLD does not only affect the liver but also associate with metabolic syndrome (MetS), type II diabetes (T2D), as well as with cardiovascular disease and chronic kidney disease (CKD). NAFLD and T2D have similar risk factors, and epidemiology and pathophysiology further emphasize their connections [5]. Evidence show that NAFLD is associated with one or more of the MetS components—arterial hypertension, central obesity, dyslipidemia, insulin resistance (IR), and T2D. The more components are present, higher are the chances for NAFLD and eventually advanced fibrosis [6]. Global NAFLD prevalence in T2D, according to meta-analysis including almost 50 thousand patients from 80 studies, was found to be as high as 55.5%. Other research showed a prevalence of NAFLD in T2D up to 59.67% and even 77.87% in obese T2D patients [7]. Remarkable, up to 5-fold increase in risk for developing T2D in patients with NAFLD was observed [8]. Once considered the hepatic manifestation of MetS, NAFLD in modern terms represents continuum from obesity to MetS and T2D [9] as there is a growing number of data suggesting that it can precede to these conditions [10]. But whether or not NAFLD is a preceding state to MetS and T2D or their consequence, it is clear that there is a vast spectrum of different signaling molecules which all interact on different levels and start a vicious self-perpetuating circle, making it hard to say what is the first “hit.” In this review, we will give comprehensive summary of pathogenesis of NAFLD focusing on insulin resistance, MetS, and T2D and their interaction as they play the central role of liver steatosis.

## 2. Pathogenesis

**Figure 1:**
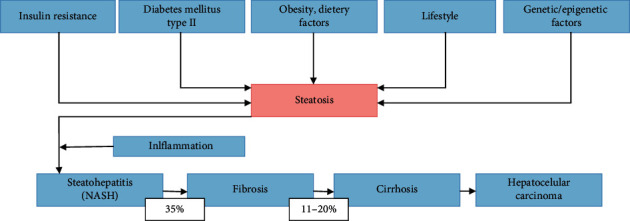
Progression of steatosis.

Pathogenesis of NAFLD is still incompletely understood as there is more than one factor contributing to this condition. Dysregulation of lipid delivery, hepatic lipid uptake, oxidation, synthesis, and secretion in very low density lipids promotes steatosis. Not all patients with liver steatosis will develop steatohepatitis, and this was initially explained by the two hit theory [11]. Certain lifestyles, combining lack of physical activity, high fat diet, and obesity, were recognized to cause steatosis as the first hit. If second hit was to occur, then it would trigger inflammation and fibrosis. Recent evidence claim the two hit hypothesis obsolete as it cannot explain multiple insults acting together on different metabolic and molecular levels [12]. Insulin resistance is just one of them, potentially most important, among other factors including adipokines such as leptin, adiponectin, resistin, gut microbiota, and other genetic, epigenetic, and environmental factors. Progression of steatosis can be seen in Figure 1.

### 2.1. Insulin Resistance

Insulin resistance plays pivotal role in liver steatosis and even more so in steatohepatitis. The term was first used almost one century ago, after the introduction of insulin therapy. Due to the low quality of the first insulin which caused production of antibodies, high doses of insulin were required, leading to overtreatment/exogenous hyperinsulinemia. Insulin resistance was defined as “a state in which a greater than normal amount of insulin is required to elicit a quantitatively normal response” [13]. Several decades later, hyperinsulinemia was recognized as an endogenous pathophysiologic mechanism, raising from insulin resistance and leading to metabolic and endocrine disruptions [14]. Today, we know that IR plays a crucial role in impaired glucose homeostasis, MetS, and T2D.

Insulin binds to the insulin receptor (a tetramer consisting of two alpha and two beta chains) on the cell surface. When insulin binds to the alpha chain, it activates a signaling cascade subsequently promoting glucose transport (glucose influx), glycogen synthesis, lipogenesis, and cell proliferation, differentiation, and survival. One the other hand, this cascade leads to downregulation of gluconeogenesis and lipolysis. The cellular insulin signaling pathway is a complex process consisting of several steps. Everything starts with autophosphorylation of the insulin receptor beta chain which activates the insulin receptor substrate (IRS 1/2). IRS activation then triggers three main pathways: PI3K/AKT (responsible for the metabolic insulin action via the translocation of the glucose transporter type 4 (GLUT4) to the plasma membrane), TSC1/2-mTOR (playing a critical role in protein synthesis since target of mTOR is a central controller for processes including RNA translation, ribosome biogenesis and autophagy, in response not only to growth factors and hormones like insulin but also to nutrients, energy, and stress signals), and RAS-MAPK pathway (promoting cell survival, division, and motility via extracellular signal-regulated kinase 1/2 (ERK1/2) complex that translocates into the nucleus activating many transcription factors, constituting an important connection between the cytoplasmic and nuclear events and orchestrating gene expression, mitogenesis, and differentiation) [15–17]. Despite greater understanding of molecular pathways in insulin signaling and metabolism, there are still numerous knowledge gaps regarding the etiology of IR in several metabolic disturbances such as NAFLD where insulin resistance seems to play crucial role.

Alterations in any of the steps in insulin signaling cascade can lead to IR, which is seen on the cellular level due to dysregulation of intracellular signals normally promoted with insulin binding. Different types of kinases and phosphatases are responsible for balancing this signaling cascade. Generally, tyrosine phosphorylation activates and serine/threonin phosphorylation inactivates insulin receptor and IRS proteins [18]. In IR, this process is mediated by several enzymes including inhibitor of kappa kinase beta (IKK-b), c-Jun-N-terminal kinase (JNK-1), and protein kinase C (PKC) which all promote serine phosphorylation of IRS and thus decrease glucose uptake, glycogen synthase activation, and also phosphorylation of forkhead box protein O (FOXO) which then result in hepatic gluconeogenesis stimulation [19, 20]. FFA, oxidative stress, and adipocyte mediating alterations are main causes of the aforementioned IKK-b, JNK-1, and PKC influences on the inhibition of IRS 1/2 signaling. Investigations also showed that inflammatory cytokines such as TNF-alpha, IL-1 beta, and IL-6 can induce serine phosphorylation of IRS1 through JNK-1, IKKb, S6K, and mTOR and induce insulin resistance [21–24]. Adipose tissue additionally plays an important role in IR as it is highly metabolic active secreting adipokines such as leptin, resistin and adiponectin. Leptin has a significant effect on IRS 1 dephosphorylation, but it is believed its role is mediated by the central nervous system rather than peripherally [25, 26]. Furthermore, leptin has a significant impact on liver fibrosis via transforming growth factor beta 1 [27]. Adiponectin levels were shown to correlate positively with insulin sensitivity, but on the other hand, it has a negative impact on inflammatory markers and TNF-alpha which induces IR, so low levels of adiponectine could potentially be significant factor of IR and lead to NAFLD [28]. Not only disruption of initiation of insulin signaling formerly explained but also termination of signaling cascade has an important role in IR. There are two most important phosphatases which terminate insulin signaling, phosphatase and tensin homolog (PTEN), and Src homology 2 domain containing inositol 5′-phosphatase 2 (SHIP2) [29]. Their increased activity terminates insulin action. The mechanism of insulin resistance is not limited to impaired insulin signaling, but it also involves the complex interplay of multiple metabolic pathways. Recent analysis of large datasets generated by metabolomics and lipidomics has revealed the role of metabolites such as lipids (saturated and unsaturated fatty acids, branched fatty acid esters of hydroxy fatty acids, diacylglycerol, sphingolipids, ceramides, and phospholipids), amino acids (methionine, circulating aromatic amino acids (AAAs) such as phenylalanine and tryptophan, branched-chain amino acids such as leucine, isoleucine, and valine), ketone bodies, and bile acids in modulating insulin sensitivity. Metabolites can regulate insulin sensitivity directly by modulating components of the insulin signaling pathway, such as insulin receptor substrates (IRSs) and AKT, and indirectly by altering the flux of substrates through multiple metabolic pathways, including lipogenesis, lipid oxidation, protein synthesis and degradation, and hepatic gluconeogenesis [30]. The aforementioned are only a part of insulin resistance etiopathology, numerous other molecular pathways in addition play an important role, and more are to be discovered in future work.

So far, it seems that excess of free fatty acids (FFA) and hyperinsulinemia are essential to start the vicious self-perpetuating circle of NAFLD. Excess FFA are in part due to increased caloric intake and obesity as well as adipocyte resistance to insulin leading to lypolysis and hyperinsulinemia. This was shown to promote lipogenesis via sterol regulatory element binding protein (SREBP1-c). SREBP1-c is just one of the lipogenesis controlling factors among others, including carbohydrate response element-binding protein (ChREBP) [31] and X-box binding protein (XBP1) otherwise known as unfolded protein response regulator (UPR). Besides controlling lipogenesis, XBP1 regulates leptin resistance, adipogenesis, inflammation, and insulin signaling and is heavily affected by endoplasmatic reticulum stress. High serum FFA, high serum cholesterol, increased lypolysis due to IR, and de novo lipogenes, decreased very low density lipoproteins (VLDL) assembly [32] all leading to high levels of liver FFA. Subsequently, this leads to lipotoxicity of accumulated fatty acids through mitochondrial dysfunction and oxidative stress as well as endoplasmatic reticulum stress. FFA normally undergo beta oxidation in the mitochondria and peroxisome as well as omega oxidation in the microsomal system. It leads not only to energy production in the form of adenosine triphosphate but also to a production of small quantities of free radicals resulting in oxidative stress [33]. Oxidative stress has been linked to the production of highly reactive intermediates during inflammation. On the other hand, reactive oxygen species (ROS) are able to further enhance the inflammatory response by triggering proinflammatory mediators (e.g., NF-kB) and cytokine production (e.g., IL-6, IL-1, IL-1*β*, IL-18, resistin, lipocalin, and TNF-alpha). The consequences of this are very dangerous, especially for nucleic acids, where modification of bases, covalent crosslinks, and single- and double-strand breaks can occur. In addition to the radical species deriving from oxygen, other radicals are derived from reactive nitrogen species (RNS), e.g., the superoxide anion (O2−) [34]. Reactive oxygen and nitrogen species (ROS) cause damage in the cell nucleus and in the mitochondria. So, in a state of IR, there is an increase in beta oxidation and thus higher ROS production. Proinflammatory factors and adipokines are relevant not only in inducing IR but also in progression of steatosis to steatohepatis. We already described how TNF-alpha has an impact on IRS1 causing IR, but, on top of that, it recruits inflammatory cells to the liver and increases reactive oxygen stress through mitochondria and promotes cell death [35]. TNF-alpha has a negative effect on adiponectin further decreasing its protective role [36]. Besides lipotoxicity, oxidative stress it causes, and adipokines dysregulation, it appears that factors such as intestinal microbiota can also play pro-inflammatory role in NAFLD and development of insulin resistance [37]. Normally, it has a role in keeping the mucosa integrity by tight junctions and has an immunomodulatory effect on innate immunity. Dysbiosis can cause increased gut permeability, and endotoxemia can occur as lipopolisaharids and can enter portal circulation and aggravate innate immune response [38]. This effect can be explained by LPS impact on toll-like receptors 4 (TRL4) and signaling pathways resulting in TNF-alpha and interleukin-1-beta (IL-1b) release [39]. Production of short chain fatty acids (SCFAs) by gut microbiota is also involved in inflammation seen by disarranged diversity and amount of SCAFs in NAFLD [40, 41].

**Figure 2:**
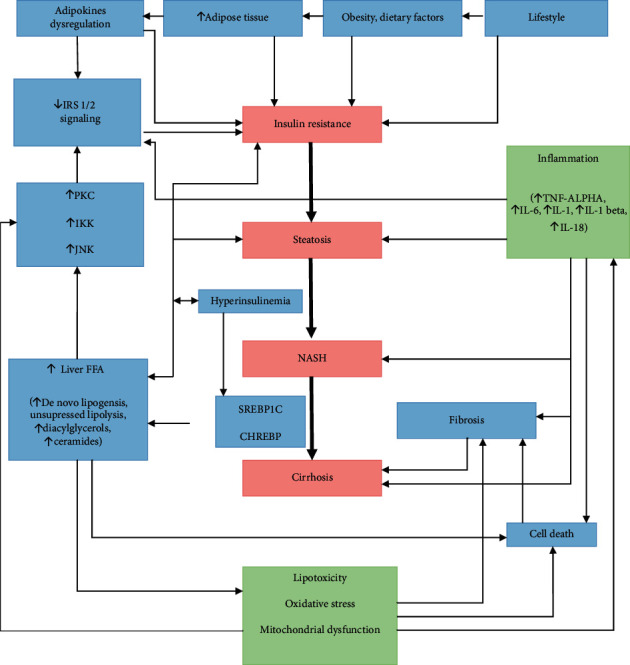
Pathophysiology.

Ultimately, lipotoxicity, inflammation, adipokine, and microbiome dysregulations will result in two main outcomes, and those are cell death and fibrosis. Hepatocyte cell death is the main trigger of progression of the disease. There are several different types of hepatocyte death, be that apoptosis, necrosis, pyroptosis, and necroptosis, but all will lead to inflammation and fibrogenesis [42]. Stellate cells are crucial in response to chronic liver injury. Activated stellate cells start to produce extracellular matrix proteins, dominantly collagen desposition. Collagen component can increase up to ten times in cirrhosis [43]. Stellate cells are not only important in fibrogenesis but also they have an inflammatory role as activated stellate cells are prone to LPS activation on the TLR4 pathway stimulating further cytokine release and activation of NK-kB and JNK pathways. All of the mechanisms we mentioned must be seen as an interactive complex happening parallel with each other (Figure 2). If the vicious circle is not stopped early, it will, in some patients, lead to cirrhosis, hepatocellular carcinoma, and liver failure as the end result of this condition. Such significant sequels will develop in up to 11–20% of NASH patients [44].

## 3. Diagnosis

NAFLD is usually discovered incidentally, by verifying elevated liver biochemical tests levels or as an incidental finding of hepatic steatosis using imaging methods. Most patients are asymptomatic (48%–100%), but some have right upper quadrant pain, fatigue, or malaise. Hepatomegaly is often seen but difficult to differentiate on physical examination because of obesity. Typical changes for chronic liver disease such as splenomegaly, spider telangiectasia, palmar erythema, and ascites are seen in patients with NASH cirrhosis. To establish the diagnosis of NAFLD, alcohol-related liver disease must be excluded, which means consumption of less than 20–40 grams of alcohol per day.

In metabolic fatty liver disease, mild to moderate elevations of serum AST or ALT level or both are recorded, usually 2- to 4-fold elevations with AST/ALT ratio <1 in most patients. The serum alkaline phosphatase level is slightly elevated in one-third of patients as well as GGT, but the serum bilirubin, serum albumin level, and prothrombin time are normal, except in patients with NAFLD-associated cirrhosis. One-fourth of patients may have ANA in low titers (<1 : 320), but other laboratory tests for other chronic liver diseases are negative. Serum and hepatic iron levels may be elevated in 20%–50% of patients with NAFLD and may be a marker of more advanced disease. A serum ferritin greater than 1.5 times the upper limit of normal has been associated with higher NAS (NAFLD Activity Score) in a study of 628 adult patients with NAFLD [45]. Clinical and laboratory findings do not correlate with the histologic severity of NAFLD, and the entire histologic spectrum of NAFLD, including cirrhosis, can be seen in patients with normal or near normal serum aminotransferase levels [46].

Imaging techniques are obtained for the evaluation of unexplained liver biochemical test abnormalities or suspected NAFLD. Ultrasound may show a “bright,” hyperechogenic liver, consistent with liver steatosis, and fatty liver can also be seen on abdominal CT or by MRI, but all these imaging methods cannot confirm the presence or determine the severity of NASH.

## 4. When to Perform Biopsy?

The reality is that most patients with NAFLD, diagnosed when hepatic steatosis is present on cross-sectional imaging studies and other chronic liver diseases are excluded, do not undergo a liver biopsy although it is required to identify patients with NASH. Liver biopsy is an invasive procedure with rare but severe complications, but it is important to differentiate patients with NASH because they are at risk of progression to cirrhosis or even HCC. That is why advanced imaging, laboratory tests, and scoring systems have been introduced to identify high-risk patients who should undergo liver biopsy.

Advanced imaging techniques include US-based technology of vibrations-controlled transient elastography (VCTE or FibroScan) which uses a low-amplitude shear wave that propagates through the liver parenchyma and magnetic resonance elastography (MRE) which combines MRI with elastography. A prospective work from Siddiqui et al. demonstrated that VCTE accurately distinguishes low from advanced stages of fibrosis but is less accurate in distinguishing intermediate stages of fibrosis or the presence of NASH [47]. MRE is excellent for staging liver fibrosis and is superior to VCTE but at higher cost and limited availability because specific MRI software and hardware are required.

Noninvasive laboratory tests have been developed to estimate the presence of steatohepatitis or fibrosis. One of them, the most promising single marker for identifying NASH is cytokeratin 18 (CK-18), a marker of apoptosis, but does it have enough sensitivity and specificity to be used alone as a predictive marker for NASH is still unknown [48, 49].

Various clinical scoring systems have also been analyzed for their ability to predict NASH or advanced fibrosis. The major clinical scoring systems include FibroTest, FibroMeter, NAFLD fibrosis score, Fibrosis-4, AST-to-platelet ratio (APRI), BARD (BMI, AST/ALT ratio, and diabetes mellitus), Enhanced Liver Fibrosis (ELF) score, NashTest, and AST/ALT ratio. Comparison of these tests in terms of positive and negative predictive values generally has demonstrated that more complicated and expensive tests are not more accurate than basic laboratory tests. These tests are good at predicting absence or advanced fibrosis and are not helpful in distinguishing intermediate stages of fibrosis [50]. NAFLD fibrosis score is the most commonly used clinical scoring algorithm that incorporates age, BMI, hyperglycaemia, AST/ALT ratio, platelet count, and serum albumin level. A low cutoff value for this score has been shown to have a high negative predictive value of 88%–93%, and a high cutoff value has shown a good positive predictive value of 82%–90%. This leaves 1 in 4 patients as having intermediate result, and for this group, a liver biopsy would be required for accurate staging.

## 5. Treatment

Currently, there is no established treatment for NAFLD or NASH. Weight loss and low-fat diet are generally recommended. There is no consensus on the most effective pharmacological agents for the treatment of NAFLD and NASH because their multifactorial pathologies are not fully understood. Histologic improvement in steatosis, inflammation, and fibrosis is the ultimate goal of treatment. Treatment strategies are now grouped into lifestyle modification, surgical interventions for weight loss, and pharmacotherapy.

### 5.1. Lifestyle Modification

Lifestyle modification includes reduction in energy intake and increase in physical activity with final goal of weight loss. Weight loss has been demonstrated to reduce liver transaminases [51–53] and decrease liver fat content. Several randomized controlled trials have shown an improvement in hepatic histology after calories intake restrictions leading to weight loss. One large prospective trial of 261 patients followed for 12 months demonstrated that all features of NASH improved with weight loss of at least 10% and fibrosis stabilized or improved with weight loss of at least 5% [54]. It has been reported that Mediterranean diet is an effective nonpharmaceutical option for diabetes type 2 and obesity [55, 56] and may improve hepatic steatosis [57], but there is no evidence that Mediterranean diet alone, without general reduction of caloric intake can be beneficial. Silymarin, the extract of milk thistle, has been used for the prevention of liver fibrosis by regulating the antifibrogenic and anti-inflammatory functions [58] and is associated with the reduction of insulin resistance and improvement in liver function [59, 60].

Omega-3 fatty acids are approved in USA for hypertriglyceridemia and have been discussed as a potential treatment for NAFLD. A meta-analysis including 355 patients demonstrated the omega-3 supplementation improved hepatic steatosis, but no histologic data were available [61]. Other research failed to show benefits, so further work is required.

### 5.2. Bariatric Surgery

Bariatric surgery is not recommended as a treatment for NAFLD and NASH, but patients who underwent bariatric surgery for other reasons had a significant weight loss that resulted in improved metabolic parameters and hepatic histology. In was reported that 85% of patients with NASH who underwent bariatric surgery had resolution of NASH and 33% had improvement in fibrosis [62] on liver biopsies one year after bariatric surgery.

### 5.3. Pharmacotherapy

Numerous drugs have been investigated for the treatment of NAFLD, and they can be grouped in weight loss medications, insulin sensitizers, antioxidants, and cytoprotective or antifibrotic agents.

### 5.4. Weight Loss Medications

The most investigated medication is orlistat, a reversible inhibitor of pancreatic and gastric lipase. It promotes weight loss through intestinal fat malabsorption. Initial trails where promising, but in the end, there was no significant weight loss between the orlistat group and placebo group [63, 64]. Side-effects, such as oily stools and potential malabsorption of other medications and reports of cholelithiasis, cholestasis, and hepatic injury, have limited the benefits of this medication.

### 5.5. Diabetic Medications

Metformin, thiazolidinediones, and incretin mimetics have been studied in the treatment of NASH. Metformin reduces plasma glucose levels primarily by reducing hepatic glucose production through the activation of AMP (adenosine monophosphate-activated protein) kinase. Activation of this enzyme also results in decreased lipid synthesis and increased fat oxidation [65]. Results have been good in mice, where metformin reduced hyperinsulinemia and improved hepatic insulin sensitivity and reduced hepatomegaly and hepatic steatosis [66], but this effect was not observed in human studies [67, 68]. Currently, metformin is not recommended for treating NAFL and NASH.

Thiazolidinediones are peroxisome proliferator-activated receptor- (PPAR-) *γ* agonists, a nuclear receptor that is expressed in adipose tissue, muscle, and liver. Rosiglitazone and pioglitazone have shown to improve insulin resistance, normalization of liver biochemical test levels, and histologic improvements [69, 70]. Meta-analysis of Musso et al. has also confirmed reducing hepatic fibrosis in patients with NASH with or without diabetes mellitus [71].

Incretin mimetics-glucaon-like-protein-1 receptor agonists (GLP-1Ras) have been shown to reduce liver inflammation and fibrosis. Furthermore, glucagon receptor agonism is being investigated for the treatment of NAFLD due to its appetite-reducing effects, as well as its ability to increase lipid oxidation and thermogenesis. Recent data suggest that glucagon receptor signaling is disrupted in NAFLD, indicating that supraphysiological glucagon receptor agonism might represent a new NAFLD treatment target. Currently available GLP-1RAs which improve insulin sensitivity and serum glucose levels promote modest weight loss and lower hepatic transaminases are exenatide, dulaglutide, semaglutide, and liraglutide [72–74]. A randomized controlled trial of 52 patients where liraglutide was compared to placebo showed significant resolution in NASH in 39% patients treated with liraglutide compared to 9% treated with placebo [75].

### 5.6. Antioxidants

Vitamin E is a potent antioxidant. It is well tolerated, improves serum aminotransferase levels, reduces hepatic steatosis, and in nondiabetics, improves steatohepatitis but not fibrosis [76, 77]. Due to cardiovascular risks in diabetic patients, vitamin E is not recommended in diabetic patients with NAFLD [78].

Carotenoids are as potent as vitamin E in inhibiting lipid peroxidation [79], but carotenoid supplementation (*β*-cryptoxanthin and astaxanthin) has not been widely used as antioxidant treatment for patients with NASH.

## 6. Conclusion

NAFLD is not an isolated condition, but a fragment of metabolic disruption emerging from high energy intake, obesity, sedentary lifestyle, and crucially IR and T2D. Prevalence of NAFLD and T2D is in remarkable rise, as both have similar risk factors, epidemiology, and pathophysiology. The presence of T2D significantly increases the chances of developing NASH and fibrosis compared to NAFLD without T2D. Relation between NAFLD and T2D is not as straightforward and these conditions have multiple interactions on different molecular levels we tried to summarize in our text. Evidence suggests that NAFLD can precede T2D, so, perhaps, by effectively managing NAFLD, we could modify the risk for T2D development in the future. Although NAFLD will eventually, in some patients, progress to liver cirrhosis and hepatocellular carcinoma, these are not the main outcomes of NAFLD as just a proportion of NASH patients will develop such significant sequels. What is more notable is the cardiovascular risk, these patients have, and cardiovascular disease are the main causes of mortality in NAFLD which further emphasizes the metabolic component of this condition. That is why screening for T2D and MetS is important when we encounter with NAFLD patients in everyday practices. The same should be done in treating patients with T2D-attention must be given to eventual concomitant liver manifestations. Unfortunately, there is no simple method in treating NAFLD and NASH, so prevention is of crucial importance. Promising multifunctional therapies are much awaited.

## Data Availability

The data used to support this study are included within article as references.

## References

[1] Chalasani N., Younossi Z., Lavine J. E. (2012). The diagnosis and management of non-alcoholic fatty liver disease: practice guideline by the American gastroenterological association, American association for the study of liver diseases, and American college of gastroenterology. *Gastroenterology*.

[2] NCD Risk Factor Collaboration (NCD-RisC) (2016). Trends in adult body-mass index in 200 countries from 1975 to 2014: a pooled analysis of 1698 population-based measurement studies with 19.2 million participants. *Lancet*.

[3] Younossi Z. M., Stepanova M., Afendy M. (2011). Changes in the prevalence of the most common causes of chronic liver diseases in the United States from 1988 to 2008. *Clinical Gastroenterology and Hepatology*.

[4] Wong R. J., Aguilar M., Cheung R. (2015). Nonalcoholic steatohepatitis is the second leading etiology of liver disease among adults awaiting liver transplantation in the United States. *Gastroenterology*.

[5] Tomah S., Alkhouri N., Hamdy O. (2020). Nonalcoholic fatty liver disease and type 2 diabetes: where do Diabetologists stand?. *Clinical Diabetes and Endocrinology*.

[6] Jinjuvadia R., Antaki F., Lohia P., Liangpunsakul S. (2017). The association between nonalcoholic fatty liver disease and metabolic abnormalities in the United States population. *Journal of Clinical Gastroenterology*.

[7] Dai W., Ye L., Liu A. (2017). Prevalence of nonal‐ coholic fatty liver disease in patients with type 2 diabetes mellitus: a meta-analysis. *Medicine*.

[8] Jäger S., Jacobs S., Kröger J. (2015). Association between the fatty liver index and risk of type 2 diabetes in the EPIC-Potsdam Study. *PLoS One*.

[9] Godoy-Matos, Amélio F., Silva Júnior W. S., Valerio C. M. (2020). NAFLD as a continuum: from obesity to metabolic syndrome and diabetes. *Diabetology & Metabolic Syndrome*.

[10] Lonardo A., Ballestri S., Marchesini G., Angulo P., Loria P. (2015). Nonalcoholic fatty liver disease: a precursor of the metabolic syndrome. *Digestive and Liver Disease*.

[11] Day C. P., James O. F. W. (1998). Hepatic steatosis: innocent bystander or guilty party?. *Hepatology*.

[12] Tilg H., Moschen A. R. (2010). Evolution of inflammation in nonalcoholic fatty liver disease: the multiple parallel hits hypothesis. *Hepatology*.

[13] Yalow R. S., Berson S. A. (1960). Plasma insulin concentrations in nondiabetic and early diabetic subjects: determinations by a new sensitive immuno-assay technic. *Diabetes*.

[14] Aronis K. N., Mantzoros C. S. (2012). A brief history of insulin resistance: from the first insulin radioimmunoassay to selectively targeting protein kinase C pathways. *Metabolism*.

[15] Haeusler R. A., McGraw T. E., Accili D. (2018). Biochemical and cellular properties of insulin receptor signalling. *Nature Reviews Molecular Cell Biology*.

[16] Boulton T. G., Nye S. H., Robbins D. J. (1991). ERKs: a family of protein-serine/threonine kinases that are activated and tyrosine phosphorylated in response to insulin and NGF. *Cell*.

[17] Di Camillo B., Carlon A., Eduati F. (2016). A rule-based model of insulin signalling pathway. *BMC Systems Biology*.

[18] Copps K. D., White M. F. (2012). Regulation of insulin sensitivity by serine/threonine phosphorylation of insulin receptor substrate proteins IRS1 and IRS2. *Diabetologia*.

[19] Polyzos S., Kountouras J., Zavos C. (2009). Nonalcoholic fatty liver disease: the pathogenetic roles of insulin resistance and adipocytokines. *Current Molecular Medicine*.

[20] Sha H., He Y., Chen H. (2009). The IRE1*α*-XBP1 pathway of the unfolded protein response is required for adipogenesis. *Cell Metabolism*.

[21] Taniguchi C. M., Emanuelli B., Kahn C. R. (2006). Critical nodes in signalling pathways: insights into insulin action. *Nature Reviews Molecular Cell Biology*.

[22] Hotamisligil G. S. (1999). Mechanisms of TNF*α*-induced insulin resistance. *Experimental and Clinical Endocrinology & Diabetes*.

[23] Xu H., Barnes G. T., Yang Q. (2003). Chronic inflammation in fat plays a crucial role in the development of obesity-related insulin resistance. *Journal of Clinical Investigation*.

[24] Osborn O., Olefsky J. M. (2012). The cellular and signaling networks linking the immune system and metabolism in disease. *Nature Medicine*.

[25] Cohen B., Novick D., Rubinstein M. (1996). Modulation of insulin activities by leptin. *Science*.

[26] Asilmaz E., Cohen P., Miyazaki M. (2004). Site and mechanism of leptin action in a rodent form of congenital lipodystrophy. *Journal of Clinical Investigation*.

[27] Leclercq I. A., Farrell G. C., Schriemer R., Robertson G. R. (2002). Leptin is essential for the hepatic fibrogenic response to chronic liver injury. *Journal of Hepatology*.

[28] Masarone M., Federico A., Abenavoli L., Loguercio C., Persico M. (2014). Non alcoholic fatty liver: epidemiology and natural history. *Reviews on Recent Clinical Trials*.

[29] Lazar D. F., Saltiel A. R. (2006). Lipid phosphatases as drug discovery targets for type 2 diabetes. *Nature Reviews Drug Discovery*.

[30] Yang Q., Vijayakumar A., Kahn B. B. (2018). Metabolites as regulators of insulin sensitivity and metabolism. *Nature Reviews Molecular Cell Biology*.

[31] Dentin R., Pégorier J.-P., Benhamed F. (2004). Hepatic glucokinase is required for the synergistic action of ChREBP and SREBP-1c on glycolytic and lipogenic gene expression. *Journal of Biological Chemistry*.

[32] Fabbrini E., Mohammed B. S., Magkos F., Korenblat K. M., Patterson B. W., Klein S. (2008). Alterations in adipose tissue and hepatic lipid kinetics in obese men and women with nonalcoholic fatty liver disease. *Gastroenterology*.

[33] Pessayre D., Berson A., Fromenty B., Mansouri A. (2001). Mitochondria in steatohepatitis. *Seminars in Liver Disease*.

[34] Roden M., Petersen K., Shulman G. (2017). Insulin resistance in type 2 diabetes. *Textbook of Diabetes*.

[35] Choi S. S., Diehl A. M. (2008). Hepatic triglyceride synthesis and nonalcoholic fatty liver disease. *Current Opinion in Lipidology*.

[36] Xu A., Wang Y., Keshaw H., Xu L. Y., Lam K. S. L., Cooper G. J. S. (2003). The fat-derived hormone adiponectin alleviates alcoholic and nonalcoholic fatty liver diseases in mice. *Journal of Clinical Investigation*.

[37] Dumas M.-E., Barton R. H., Toye A. (2006). Metabolic profiling reveals a contribution of gut microbiota to fatty liver phenotype in insulin-resistant mice. *Proceedings of the National Academy of Sciences*.

[38] Day C. P., James O. F. W. (1998). Steatohepatitis: a tale of two “hits”?. *Gastroenterology*.

[39] Yu Y., Cai J., She Z., Li H. (2019). Insights into the epidemiology, pathogenesis, and therapeutics of nonalcoholic fatty liver diseases. *Advanced Science*.

[40] Abu-Shanab A., Quigley E. M. M. (2010). The role of the gut microbiota in nonalcoholic fatty liver disease. *Nature Reviews Gastroenterology & Hepatology*.

[41] Saad M. J. A., Santos A., Prada P. O. (2016). Linking gut microbiota and inflammation to obesity and insulin resistance. *Physiology*.

[42] Marra F., Gastaldelli A., Svegliati Baroni G., Tell G., Tiribelli C. (2008). Molecular basis and mechanisms of progression of non-alcoholic steatohepatitis. *Trends in Molecular Medicine*.

[43] Schuppan D. (1990). Structure of the extracellular matrix in normal and fibrotic liver: collagens and glycoproteins. *Seminars in Liver Disease*.

[44] Singh S., Allen A. M., Wang Z. (2015). Fibrosis progression in nonalcoholic fatty liver vs. nonalcoholic steatohepatitis: a systematic review and meta-analysis of paired-biopsy studies. *Clinical Gastroenterology and Hepatology*.

[45] Kowdley K. V., Belt P., Wilson L. A. (2012). Serum ferritin is an independent predictor of histologic severity and advanced fibrosis in patients with nonalcoholic fatty liver disease. *Hepatology*.

[46] Verma S., Jensen D., Hart J., Mohanty S. R. (2013). Predictive value of ALT levels for non-alcoholic steatohepatitis (NASH) and advanced fibrosis in non-alcoholic fatty liver disease (NAFLD). *Liver International*.

[47] Siddiqui M. S., Vuppalanchi R., Van Nattaetal M. L. (2019). Vibration-controlled transient elastography to asses fibrosis and steatosis in patients with non-alcoholich fatty liver disease. *Clinical Gastroenterology and Hepatology*.

[48] Feldstein A. E., Wieckowska A., Lopez A. R., Liu Y.-C., Zein N. N., McCullough A. J. (2009). Cytokeratin-18 fragment levels as noninvasive biomarkers for nonalcoholic steatohepatitis: a multicenter validation study. *Hepatology*.

[49] Cusi K., Chang Z., Harrison S. (2014). Limited value of plasma cytokeratin-18 as a biomarker for NASH and fibrosis in patients with non-alcoholic fatty liver disease. *Journal of Hepatology*.

[50] Vilar-Gomez E., Chalasani N. (2018). Non-invasive assessment of non-alcoholic fatty liver disease: clinical prediction rules and blood-based biomarkers. *Journal of Hepatology*.

[51] Park H. S., Kim M. W., Shin E. S. (1995). Effect of weight control on hepatic abnormalities in obese patients with fatty liver. *Journal of Korean Medical Science*.

[52] Ueno T., Sugawara H., Sujaku K. (1997). Therapeutic effects of restricted diet and exercise in obese patients with fatty liver. *Journal of Hepatology*.

[53] Suzuki A., Lindor K., Saver J. S. (2005). Effect of changes on body weight and lifestyle in nonalcoholic fatty liver disease. *Journal of Hepatology*.

[54] Vilar-Gomez E., Martinez-Perez Y., Calzadilla-Bertot L. (2015). Weight loss through lifestyle modification significantly reduces features of nonalcoholic steatohepatitis. *Gastroenterology*.

[55] Martínez-González M. Á., Fuente-Arrillaga C. D. L., Nunez-Cordoba J. M. (2008). Adherence to Mediterranean diet and risk of developing diabetes: prospective cohort study. *British Medical Journal*.

[56] Romaguera D., Norat T., Mouw T. (2009). Adherence to the Mediterranean diet is associated with lower abdominal adiposity in European men and women. *The Journal of Nutrition*.

[57] Haufe S., Engeli S., Kast P. (2011). Randomized comparison of reduced fat and reduced carbohydrate hypocaloric diets on intrahepatic fat in overweight and obese human subjects. *Hepatology*.

[58] Cacciapuoti F., Scognamiglio A., Palumbo R., Forte R. (2013). Silymarin in non alcoholic fatty liver disease. *World Journal of Hepatology*.

[59] Velussi M., Cernigoi A. M., Ariella D. M., Dapas F., Caffau C., Zilli M. (1997). Long-term (23 months) treatment with an anti-oxidant drug (silymarin) is effective on hyperinsulinemia, exogenous insulin need and malondialdehyde levels in cirrhotic diabetic patients. *Journal of Hepatology*.

[60] Hajaghamohammadi A. A., Ziaee A., Raflei R. (2008). The efficacy of silymarin in decreasing transaminase activities in nonalcoholic fatty liver disease: a randomized controlled clinical trial. *Hepatitis Monthly*.

[61] Parker H. M., Johnson N. A., Burdon C. A., Cohn J. S., O’Connor H. T., George J. (2012). Omega-3 supplementation and non-alcoholic fatty liver disease: a systematic review and meta-analysis. *Journal of Hepatology*.

[62] Lassailly G., Caiazzo R., Buob D. (2015). Bariatric surgery reduces features of nonalcoholic steatohepatitis in morbidly obese patients. *Gastroenterology*.

[63] Zelber-Sagi S., Kessler A., Brazoswky E. (2006). A double-blindrandomizedplacebocontrolledtrial od orlistat for treatmentofnonalcoholicfattyliverdisease. *Clinical Gastroenterology and Hepatology*.

[64] Harrison S. A., Fecht W., Brunt E. M., Neuschwander-Tetri B. A. (2009). Orlistat for overweight subjects with nonalcoholic steatohepatitis: a randomized, prospective trial. *Hepatology*.

[65] Zhou G., Myers R., Li Y. (2001). Role of AMP-activated protein kinase in mechanism of metformin action. *Journal of Clinical Investigation*.

[66] Lin H. Z., Yang S. Q., Chuckaree C., Kuhajda F., Ronnet G., Diehl A. M. (2000). Metformin reverses fatty liver disease in obese, leptin-deficient mice. *Nature Medicine*.

[67] Bugianesi E., Gentilcore E., Manini R. (2005). A randomized controlled trial of metformin versus vitamin E or prescriptive diet in nonalcoholic fatty liver disease. *The American Journal of Gastroenterology*.

[68] Haukeland J. W., Konopski Z., Eggesbø H. B. (2009). Metformin in patients with non-alcoholic fatty liver disease: a randomized, controlled trial. *Scandinavian Journal of Gastroenterology*.

[69] Belfort R., Harrison S. A., Brown K. (2006). A placebo-controlled trial of pioglitazone in subjects with nonalcoholic steatohepatitis. *New England Journal of Medicine*.

[70] Ratziu V., Charlotte F., Bernhardt C. (2010). Long-term efficacy of rosiglitazone in nonalcoholic steatohepatitis: results of the fatty liver improvement by rosiglitazone therapy (FLIRT 2) extension trial. *Hepatology*.

[71] Musso G., Cassader M., Paschetta E., Gambino R. (2017). Thiazolidinediones and advanced liver fibrosis in nonalcoholic steatohepatitis. *JAMA Internal Medicine*.

[72] Seghieri M., Christensen A. S., Andersen A., Solini A., Knop F. K., Vilsbøll T. (2018). Future perspectives on GLP-1 receptor agonists and GLP-1/glucagon receptor co-agonists in the treatment of NAFLD. *Frontiers in Endocrinology*.

[73] Seko Y., Sumida Y., Tanaka S. (2017). Effect of 12-week dulaglutide therapy in Japanese patients with biopsy-proven non-alcoholic fatty liver disease and type 2 diabetes mellitus. *Hepatology Research*.

[74] Newsome P., Francque S., Harrison S. (2019). Effect of semaglutide on liver enzymes and markers of inflammation in subjects with type 2 diabetes and/or obesity. *Alimentary Pharmacology & Therapeutics*.

[75] Armstrong M. J., Gaunt P., Aithal G. P. (2016). Liraglutide safety and efficacy in patients with non-alcoholic steatohepatitis (LEAN): a multicentre, double-blind, randomised, placebo-controlled phase 2 study. *The Lancet*.

[76] Harrison S. A., Torgerson S., Hayashi P., Ward J., Schenker S. (2003). Vitamin E and vitamin C treatment improves fibrosis in patients with nonalcoholic steatohepatitis. *The American Journal of Gastroenterology*.

[77] Sato K., Gosho M., Yamamoto T. (2015). Vitamin E has a beneficial effect on nonalcoholic fatty liver disease: a meta-analysis of randomized controlled trials. *Nutrition*.

[78] Clarke M. W., Burnett J. R., Croft K. D. (2008). Vitamin E in human health and disease. *Critical Reviews in Clinical Laboratory Sciences*.

[79] Rock C. L., Jacob R. A., Bowen P. E. (1996). Update on the biological characteristics of the antioxidant micronutrients. *Journal of the American Dietetic Association*.

